# Hypopigmentation and Maternal-Zygotic Embryonic Lethality Caused by a Hypomorphic *Mbtps1* Mutation in Mice

**DOI:** 10.1534/g3.112.002196

**Published:** 2012-04-01

**Authors:** Sophie Rutschmann, Karine Crozat, Xiaohong Li, Xin Du, Jeffrey C. Hanselman, Alana A. Shigeoka, Katharina Brandl, Daniel L. Popkin, Dianne B. McKay, Yu Xia, Eva Marie Y. Moresco, Bruce Beutler

**Affiliations:** * Department of Genetics and; ‡ Department of Immunology and Microbial Science, The Scripps Research Institute, La Jolla, California 92037; † Pfizer Global Research and Development, Department of Cardiovascular Pharmacology, Ann Arbor, Michigan 48105

**Keywords:** pigmentation, coat color, site 1 protease, cholesterol, maternal-zygotic effect lethality

## Abstract

The site 1 protease, encoded by *Mbtps1*, mediates the initial cleavage of site 2 protease substrates, including sterol regulatory element binding proteins and CREB/ATF transcription factors. We demonstrate that a hypomorphic mutation of *Mbtps1* called *woodrat* (*wrt*) caused hypocholesterolemia, as well as progressive hypopigmentation of the coat, that appears to be mechanistically unrelated. Hypopigmentation was rescued by transgenic expression of wild-type *Mbtps1*, and reciprocal grafting studies showed that normal pigmentation depended upon both cell-intrinsic or paracrine factors, as well as factors that act systemically, both of which are lacking in *wrt* homozygotes. *Mbtps1* exhibited a maternal-zygotic effect characterized by fully penetrant embryonic lethality of maternal-zygotic *wrt* mutant offspring and partial embryonic lethality (~40%) of zygotic *wrt* mutant offspring. *Mbtps1* is one of two maternal-zygotic effect genes identified in mammals to date. It functions nonredundantly in pigmentation and embryogenesis.

The site 1 protease (S1P), a transmembrane serine protease also known as subtilisin-kexin-isoenzyme 1, operates within the Golgi apparatus, where it performs the initial extracytoplasmic cleavage of site 2 protease (S2P) substrates during regulated intramembrane proteolysis, a process by which transmembrane proteins are cleaved within a membrane-spanning helix to release cytosolic domains (Ehrmann and Clausen 2004). S1P cleaves the sterol regulatory element binding proteins (SREBPs) to regulate cholesterol homeostasis; CREB/ATF transcription factors ATF6 (Ye *et al.* 2000), CREBH (Zhang *et al.* 2006), CREB4 (Stirling and O’hare 2006), OASIS (Murakami *et al.* 2006), and Luman (Raggo *et al.* 2002) in response to endoplasmic reticulum (ER) stress; and the surface glycoprotein precursors of lymphocytic choriomeningitis virus, Lassa fever virus, and Crimean-Congo hemorrhagic fever virus (Beyer *et al.* 2003; Kunz *et al.* 2003; Lenz *et al.* 2001; Vincent *et al.* 2003).

In the mouse, targeted disruption of the S1P-encoding gene *Mbtps1* prevented normal epiblast formation and subsequent implantation of the embryo (Mitchell *et al.* 2001), resulting in lethality at an early developmental stage (Yang *et al.* 2001). Liver-specific knockout of *Mbtps1* yielded viable mice in which blood cholesterol and triglyceride levels and the expression of genes involved in fatty acid and cholesterol synthesis and low-density lipoprotein (LDL) clearance were affected (Yang *et al.* 2001). Cartilage-specific *Mbtps1*-deficient mice died shortly after birth, exhibiting severe chondrodysplasia caused by the retention of type 2 collagen in the ER (Patra *et al.* 2007).

We recently generated a viable hypomorphic allele of *Mbtps1*, called *woodrat*, with the substitution Y496C in the extracellular domain. Homozygosity for the *woodrat* mutation caused hypersensitivity to dextran sodium sulfate−induced colitis by impairing the unfolded protein response in intestinal epithelial cells (Brandl *et al.* 2009) and conferred resistance to lymphocytic choriomeningitis virus through an effect on dendritic cells (Popkin *et al.* 2011). We demonstrate here that cell-intrinsic and -extrinsic functions of S1P are required for normal coat color. In addition, the *wrt* mutation of *Mbtps1* causes maternal-zygotic effect embryonic lethality.

## Materials and Methods

### Mice

Mice were maintained in specific pathogen-free conditions in The Scripps Research Institute Vivarium under the supervision of the Department of Animal Resources. Animals were fed a normal (Teklad #7012; Harlan) or a 5% cholesterol-enriched diet (TD00337; Harlan). All experimental procedures were conducted in accordance with institutional guidelines for animal care and use. C57BL/6J mice were purchased from The Jackson Laboratories. *Tyr^c-ghost^* mutant mice were produced and maintained in the Beutler lab. The *Mbtps1^wrt/wrt^* strain was generated by *N*-ethyl-*N*-nitrosourea mutagenesis of C57BL/6J mice (Brandl *et al.* 2009) and maintained by breeding *Mbtps1^wrt/wrt^* males to *Mbtps1^wrt/^*^+^ females. The *Tyr^c-ghost^* and *Mbtps1^wrt^* strains are described at http://mutagenetix.utsouthwestern.edu.

For phenotypic rescue experiments, a BAC (CH29-174A18) containing the wild-type allele of *Mbtps1* derived from the NOD/LtJ strain was injected into the male pronucleus of fertilized oocytes from matings of C57BL/6J females and *Mbtps1^wrt/wrt^* males. The limits of the genomic DNA sequence included in the BAC are 8:121841017−122109039. The expression of the transgenic wild-type *Mbtps1* allele in mice was confirmed by detection via polymerase chain reaction (PCR) of a GATA simple sequence length polymorphism present in the BAC and informative for C57BL/6J *vs.* NOD/LtJ strains using the following primers: 5′-CCAGCGGTTAATGGCATCTGAAATG-3′ and 5′-ATTGTCCTAAGCTGGGTGGCAGAG-3′.

### Real-time PCR analysis

RNA from skin was isolated using the Trizol reagent (Invitrogen). DNAse-treated RNA underwent randomly primed cDNA synthesis and real-time PCR analysis. SYBR Green-based real-time PCR was performed using the DyNAmo SYBR Green qPCR Kit (Finnzymes). Mitf-specific primers were obtained from QIAGEN, and signals were normalized to β-actin. Normalized data were used to quantitate relative levels of Mitf using ΔΔCt analysis.

### Skin grafts

Recipient mice were anesthetized and the flank hair shaved with electronic clippers. A graft bed was prepared on the lateral thoracic region under aseptic conditions. The graft bed was prepared by carefully removing the epidermis and dermis to the level of the panniculus carnosus without disturbing the vascular bed. Donor thoracic skin was prepared in the same manner, *i.e.*, removing the epidermis and dermis and placing in a sterile Petri dish wetted with phosphate-buffered saline. The donor skin was then placed into the recipient vascular bed and a 1- to 2-mm margin left on all sides. The grafted skin was covered with sterile, antibiotic (bacitracin)-impregnated Vaseline gauze, covered with a bandage, and then wrapped in cloth tape. The grafts were left undisturbed for 7 days. On day 7, the bandages were removed, and the grafts were photographed on a daily basis.

### Cholesterol, triglycerides, high-density lipoprotein cholesterol, LDL-cholesterol, and very high-density lipoprotein cholesterol measurement

Four mice per genotype were fasted for 4 hr before blood collection from the retro-orbital sinus. Serum concentrations of total cholesterol and triglycerides were determined enzymatically on a Cobas Mira Plus autoanalyzer using the cholesterol R1 and triglycerides reagent methods, respectively (Roche Diagnostics). Colorimetric changes were measured at 500 nm. Lipoprotein-associated cholesterol was separated using the SPIFE 3000 agarose electrophoresis system (Helena Labs, Beaumont, TX) (Contois *et al.* 1999) and verified by fast-protein liquid chromatography with a Superose 6HR column and in-line post column analysis as described previously (Kieft *et al.* 1991).

### Analysis of SREBP1 and SREBP2 processing

For SREBP analysis, livers were collected from mice fasted for 4 hr and homogenized in a Potter-Elvehjem tissue grinder in a buffer containing 150 mM NaCl, 0.5% NP-40, 10 mM Tris, pH 7.4, and 1 mM EDTA and complete protease inhibitor cocktail (Roche Biochemicals). The cell membrane-containing pellet obtained by centrifugation at 14,000 rpm for 5 min at 4° was vortexed at 4° in a second buffer made of 20 mM HEPES, pH 7.9, 25% glycerol, 0.4 M NaCl, 1 mM EDTA, 1 mM EGTA, and complete protease inhibitor cocktail (Roche Biochemicals). The supernatant obtained from a 5-min centrifugation at 14,000 rpm at 4° was resuspended in sodium dodecyl sulfate-polyacrylamide gel electrophoresis loading buffer (0.5 M dithiothreitol, 10% sodium dodecyl sulfate, 1 M Tris, pH 6.8, 50% glycerol, 0.2% bromophenol blue powder). Proteins were separated by gel electrophoresis and transferred to a nitrocellulose membrane. After blocking in 5% skim milk powder in phosphate-buffered saline, membranes were incubated with rabbit anti-SREBP1 or anti-SREBP2 primary antibodies (Affinity BioReagents), followed by a secondary goat anti-rabbit IgG antibody coupled to peroxidase (Rockland). Blots were stained with Rouge Ponceau (Sigma-Aldrich) and then developed by enhanced chemiluminescence using Super Signal West Pico ECL Substrate (Pierce); signals were recorded on autoradiographic film (Kodak, Rochester, NY). Precursors SREBPs (~120 kD) were distinguished from processed SREBPs by size (~78 kD).

### Statistical analysis

The significance of the outcomes of crosses was calculated using the χ^2^ test on the basis of the expected frequencies of hypopigmented (*Mbtps1^wrt/wrt^*) and normal (*Mbtps1^+/+^* and /or *Mbtps1^wrt/+^*) progeny from a given cross, unless otherwise specified. The statistical significance of all other differences was determined by a two-tailed Student’s *t*-test. Differences with a *P* value <0.05 were considered statistically significant. All error bars represent SD.

## Results

### S1P is required for normal coat color

**Figure 1:**
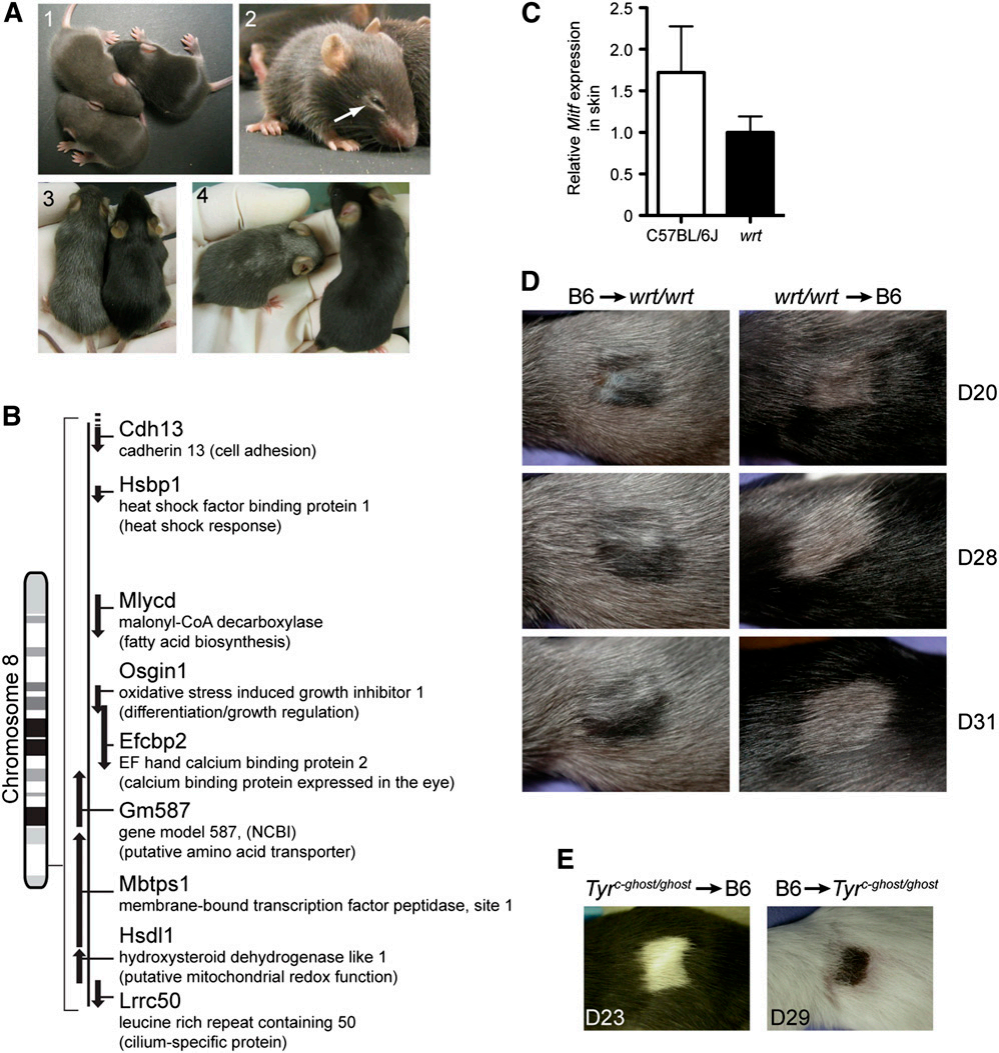
The *woodrat* mutation affects coat color in both a cell-autonomous and systemic manner. (A) The *woodrat* coat color phenotype at 8 days (1), 15 days (2), 18 days (3), and 6 weeks of age (4). (B) Chromosome 8 genes encompassed by the BAC used for transgenesis. (C) Relative expression of *Mitf* mRNA measured in the skin of wild type and *Mbtps1^wrt/wrt^* mice by reverse transcription PCR. (D) Skin grafted from a C57BL/6J (B6) mouse onto a *Mbtps1^wrt/wrt^* mouse and from a *Mbtps1^wrt/wrt^* mouse onto a C57BL/6J mouse on the days indicated posttransplantation. Results are representative of 3 (*wrt/wrt*→C57BL/6J) transplants and 3 (C57BL/6J→*wrt/wrt*) transplants. (E) Skin grafted from a *Tyr^c-ghost^* homozygous mutant mouse onto a wild-type C57BL/6J mouse and from a wild-type C57BL/6J mouse onto a *Tyr^c-ghost^* homozygote.

The recessive *woodrat* (*wrt*) mutation was isolated by positional cloning based on a hypopigmentation phenotype (Brandl *et al.* 2009). Mild uniform hypopigmentation was first observed in *Mbtps1^wrt/wrt^* homozygotes at 8 days of age (Figure 1A). Starting from a white ring around the eyes, the phenotype progressed irregularly to produce a coat composed of a combination of white and black hairs. In adults, the phenotype stabilized as a homogenous coat consisting of hairs with alternately normal or absent pigmentation.

To confirm that the *Mbtps1^wrt^* mutation was responsible for the observed progressive hypopigmentation, we tested whether the phenotype could be rescued by transgenic expression of wild-type *Mbtps1* in homozygous *woodrat* mice. A bacterial artificial chromosome (BAC) containing a segment of chromosome 8 genomic DNA encompassing the *Mbtps1* locus (Figure 1B) was injected into the male pronucleus of fertilized C57BL/6J oocytes, from which 46 pups were born, seven of which carried the transgene. Genomic DNA from a *Mbtps1^wrt/wrt^* mouse was sequenced extensively over the region included within the BAC, so that 92.4% of all nucleotides comprising coding regions and splice junctions (defined as 10 bp at either end of each intron) were examined on at least one strand to a phred score of 30 or greater. No mutations other than the *Mbtps1^wrt^* allele were detected.

Two independent transgenic founders, both female heterozygotes, were crossed to male *Mbtps1^wrt/wrt^* mice and the heterozygous progeny were crossed to the parental *Mbtps1^wrt/wrt^* strain to achieve homozygosity for the *wrt* allele in a fraction of the offspring, 50% of which would be expected to inherit the transgene. Nine of them showed the hypopigmentation phenotype, all were homozygous for the *wrt* allele and none carried a transgene (*P* = 0.00195; exact binomial probability). Nineteen offspring carried a transgene, derived from one founder or the other, and all showed a wild-type coat color (*P* = 0.00000191; exact binomial probability). Combining these probabilities by Fisher’s method, the null hypothesis that the transgene does not affect the *woodrat* phenotype is rejected (χ^2^ on 4 df = 38.8; *P* < 0.0001).

Mitf is a key transcriptional regulator of melanocyte development, and several mutant alleles of *Mitf*, including *mi-enu5*, *mi-bcc2* (Hansdottir *et al.* 2004), and *mi-vit* (Boissy *et al.* 1987; Lerner 1986), cause hypopigmentation similar in various aspects to that observed in *wrt* homozygotes. However, we found no significant difference between *Mitf* mRNA expression in the skin of wild-type *vs.* *Mbtps1^wrt/wrt^* mice (Figure 1C). Consistent with this finding, melanocytes of normal morphological appearance were present in the hair follicles of *Mbtps1^wrt/wrt^* mice; however, the functional status of those melanocytes was not evaluated. These findings suggest that the hypopigmentation of *Mbtps1^wrt/wrt^* mice is not caused by aberrant melanocyte development.

To determine whether the *wrt* pigmentation phenotype resulted from a systemic metabolic defect or from a defect localized to the skin, syngeneic skin transplants were performed (Figure 1D). Shaved dorsal skin from *Mbtps1^wrt/wrt^* or C57BL/6J mice was grafted onto C57BL/6J or *Mbtps1^wrt/wrt^* mice, respectively. In a wild-type C57BL/6J animal, *Mbtps1^wrt/wrt^* grafts retained their hypopigmented phenotype, whereas C57BL/6J skin transplanted onto a *Mbtps1^wrt/wrt^* individual acquired a hypopigmented phenotype. Control C57BL/6J skin grafts maintained their black color when transplanted onto a *Tyr^c-ghost^* homozygous mutant mouse, and reciprocal transplantation of skin from the *Tyr^c-ghost^* homozygous mutant onto wild-type recipients remained white (Figure 1E). These results indicate that normal pigmentation depends upon two independent processes, one systemic and one cell-autonomous or paracrine, both of which are disrupted by homozygosity for the *Mbtps1^wrt^* mutation.

### Maternal-zygotic effect lethality caused by the *woodrat* mutation

Homozygosity for the *wrt* allele biases against survival *in utero*. Of 87 progeny in 23 litters born from crosses of *Mbtps1^wrt/wrt^* males to *Mbtps1^wrt/+^* females, in which an equal number of homozygous and heterozygous progeny would be expected, 60 phenotypically normal and 27 phenotypically affected mice were produced (*P* = 0.0004). When fetuses from this cross were examined at 11 (n = 7 embryos, 1 litter), 14 (n = 6 embryos, 1 litter), 17 (n = 8 embryos, 1 litter), and 20 days of gestation (n = 6 embryos, 1 litter), 100% were found alive (Table 1), suggesting that death of homozygotes occurred before embryonic day 11. In independent crosses of heterozygotes, among 18 litters, 90 phenotypically normal and 17 phenotypically affected mice were produced (*P* = 0.0295). All litters were carefully counted on the day of birth, and no postnatal deaths were observed before genotyping at 19 days of age.

**Table 1:** Viability of embryos with the *wrt* mutation

Parents	Gestation Period (d)	Litters (no.)	Total Embryos (no.)	
			Alive	Dead
♂*Mbtps^wrt/wrt^* × ♀*Mbtps^wrt/^*^+^	11	1	7	0
	14	1	6	0
	17	1	8	0
	20	1	6	0
♂*Mbtps^wrt/wrt^* × ♀*Mbtps^wrt/wrt^*	0.5	2	9	0
	8	1	0	0
	11	2	0	0
	14	2	0	0

We also observed that no offspring were born from crosses of *Mbtps1^wrt/wrt^* males and *Mbtps1^wrt/wrt^* females; these female mice were surveyed daily from day 18 to day 24 after observation of the copulatory plug. *Mbtps1^wrt/wrt^* females crossed to *Mbtps1^wrt/+^* males or *Mbtps1^+/+^* males gave birth to heterozygous pups, but in the former cross, never to homozygous pups; litters were counted on the day of birth and deaths among pups were not observed before genotyping at postnatal day 19. However, as already noted, *Mbtps1^wrt/+^* females crossed to *Mbtps1^wrt/wrt^* males produced both heterozygous and homozygous offspring. Thus, the *wrt* allele causes maternal-zygotic effect lethality in that homozygous females can only give birth to offspring carrying at least one wild-type copy of the *Mbtps1* locus, but heterozygous females that carry one wild-type copy of *Mbtps1* can give birth to offspring lacking wild type *Mbtps1*.

**Figure 2:**
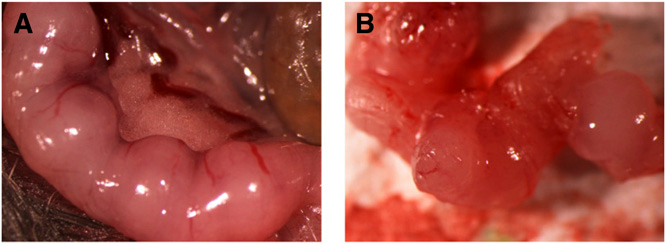
Embryonic lethality of homozygous maternal-zyogtic *wrt* mutants before 8 days of gestation. (A) The uterus of a pregnant *Mbtps1^wrt/wrt^* female mated to a *Mbtps1^wrt/wrt^* male on the eighth day of gestation appeared to contain embryos. (B) However, no fetal bodies were found inside it.

To determine the stage at which development of homozygous maternal-zygotic mutants fails, embryos from *Mbtps1^wrt/wrt^* females crossed to *Mbtps1^wrt/wrt^* males were examined after various gestation periods. *Mbtps1^wrt/wrt^* females crossed to *Mbtps1^wrt/wrt^* males appeared pregnant 8 days postcoitum and left and right uterine horns were highly vascularized and displayed rounded bulges (Figure 2A). However, no fetal bodies were found within their uteri at 8, 11, or 14 days of gestation (Figure 2B and Table 1). Thus, death and resorption of maternal-zygotic mutant embryos (homozygous *wrt* embryos derived from homozygous *wrt* mothers) occur before the eighth day of gestation.

### Low cholesterol levels in *wrt* homozygotes are not responsible for hypopigmentation

**Figure 3:**
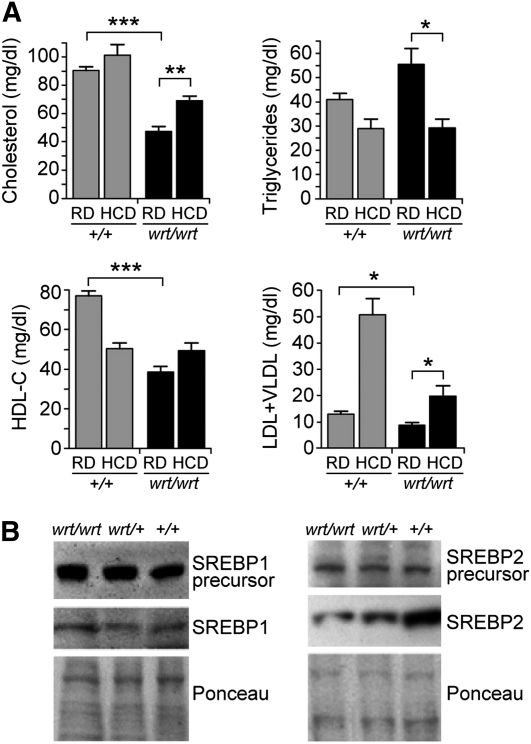
Reduced serum cholesterol in *Mbtps1^wrt/wrt^* mice. (A) Cholesterol, triglyceride, HDL-C, and LDL-C/VLDL-C concentrations in the serum of 8-week-old *Mbtps1^+/+^* and *Mbtps1^wrt/wrt^* mice fed a regular diet (RD) or a diet supplemented with 5% cholesterol (HCD) for 1 week. Each bar represents the average for four mice. Error bars represent SD. ^*^P < 0.05; ^**^P < 0.01; ^***^P < 0.001. (B) SREBP1 and SREBP2 cleavage in *Mbtps1^wrt/wrt^*, *Mbtps1^wrt/+^*, and *Mbtps1^+/+^* hepatocytes after 4 hr of fasting. Protein loading was assessed with Ponceau Red. Results are representative of four experiments for SREBP1 and three experiments for SREBP2, each using three mice per genotype.

Because S1P has nonredundant functions in both cholesterol and triglyceride homeostasis through cleavage of SREBPs (Sakai *et al.* 1998), we measured the levels of cholesterol, triglyceride, high-density lipoprotein cholesterol (HDL-C), and low-density lipoprotein-C (LDL-C)/very low-density lipoprotein-C (VLDL-C) in *Mbtps1^wrt/wrt^* mice fasted for 4 hr (Figure 3A). Serum concentrations of cholesterol and lipoproteins were significantly reduced in homozygous mutant mice as compared to wild-type controls. Triglyceride levels were not affected by the *wrt* mutation. To determine whether a high-cholesterol diet could rescue the low levels of serum cholesterol, HDL-C, and LDL-C/VLDL-C, as well as the pigmentation defect in *wrt* homozygotes, mice were fed a high-cholesterol diet and observed for 1 week. The high-cholesterol diet increased the concentrations of total cholesterol and LDL-C/VLDL-C in the serum of *Mbtps1^wrt/wrt^* mice relative to levels in *Mbtps1^wrt/wrt^* mice fed a regular diet (Figure 3A). However, the high-cholesterol diet did not change the abnormal coat pigmentation of *Mbtps1^wrt/wrt^* mice.

There are three isoforms of SREBP. SREBP2 preferentially up-regulates the expression of genes involved in cholesterol synthesis, whereas SREBP1c selectively induces lipogenic genes without affecting cholesterol synthesis genes (Eberle *et al.* 2004), and SREBP1a leads to accumulation of both cholesterol and triglycerides. We hypothesized that the disproportionate effect of the *woodrat* mutation on serum cholesterol *vs.* triglyceride concentration might reflect substrate specificity, *i.e.*, more efficient regulated intramembrane proteolysis processing of SREBP1c relative to SREBP2 in *Mbtps1^wrt/wrt^* mice. Consistent with this hypothesis, we observed that whereas the SREBP1 precursor was equally processed into its 68 kD active form in hepatocytes from *Mbtps1^wrt/wrt^*, *Mbtps1^wrt/+^*, and wild-type mice, a reduced amount of precursor SREBP2 was processed into its cleaved, active form in *Mbtps1^wrt/wrt^* compared with *Mbtps1^wrt/+^* or wild-type hepatocytes (Figure 3B).

## Discussion

In previous studies, we established that a gene trap allele of *Mbtps1* was lethal both in the homozygous state and in *trans* with *Mbtps1^wrt^* (Brandl *et al.* 2009). However, although hypopigmentation was the phenotype used to map the *Mbtps1^wrt^* mutation, no firm conclusion could be drawn concerning cause and effect. Using transgenesis, we have now adduced strong evidence that the previously reported coat color anomaly of the *wrt* strain does, in fact, result from the hypomorphic *wrt* mutation of *Mbtps1*. In a compound heterozygous cross, *Mbtps1^wrt/wrt^* mice that were also transgenic for the wild-type *Mbtps1* locus never displayed hypopigmentation and all mice that did display hypopigmentation were of the *Mbtps1^wrt/wrt^* genotype, and lacked the transgene.

More than 300 gene products are known to affect mouse pigmentation (Montoliu *et al.* 2011), including proteins that regulate melanocyte proliferation and development (*e.g.*, Mitf, Kit, Edn1), melanosome biogenesis (*e.g.*, Oca2, Slc45a2, BLOC-1 complex), melanosome transport (*e.g.*, Rab27a, melanophilin, myosin Va), and melanogenesis (*e.g.*, Tyr, Dct, Mc1r). Most classical coat color genes encode factors produced in the skin and/or hair follicle that signal locally to melanocytes or within them (Hirobe 2011; Slominski *et al.* 2004). In general, less is known of factors that circulate through the bloodstream and exert systemic control over pigmentation. One well-studied example is α-melanocyte−signaling hormone, which is produced in humans by melanocytes and keratinocytes (Chakraborty *et al.* 1996; Rousseau *et al.* 2007; Wakamatsu *et al.* 1997) and in mice and humans by the pituitary gland (Hirobe *et al.* 2004; Krude *et al.* 1998; Pears *et al.* 1992), through cleavage of the precursor protein proopiomelanocortin. α-melanocyte−signaling hormone from either tissue source in humans, and from the pituitary gland via the bloodstream in mice, promotes the production of eumelanin by melanocytes. Other systemic factors influencing pigmentation include steroid hormones (*e.g.*, estrogen, progesterone, androgen), fatty acids (*e.g.*, linoleic acid, palmitic acid), and iron (Hirobe 2011).

Using reciprocal immunologically compatible skin grafts, we showed that normal pigmentation of the fur depends upon two processes, one cell-autonomous or paracrine and one systemic, both of which are disrupted by homozygosity for the *wrt* mutation. The effects of *Mbtps1* activity on cells or tissues at remote locations may be interpreted in several ways. First, it has been noted that both an ER/Golgi membrane-anchored and a shed, soluble form of S1P exist, and the soluble form might conceivably exert a systemic effect on pigmentation. Second, it may be imagined that specific metabolites, dependent upon the enzymatic activity of S1P cleavage products, might be needed for this process. Although we showed that serum cholesterol levels were reduced in *Mbtps1^wrt/wrt^* mice, cholesterol does not seem to be the crucial metabolite needed for normal pigmentation, since a high cholesterol diet did not normalize the coat pigmentation of *Mbtps1^wrt/wrt^* mice.

In breeding experiments, maternal-zygotic *Mbtps1^wrt^* mutant offspring (homozygotes derived from homozygous mutant mothers) displayed fully penetrant embryonic lethality, whereas zygotic mutant offspring (homozygotes derived from heterozygous mothers) displayed partial (~40%) embryonic lethality, and heterozygous mutant offspring of homozygous mothers were fully viable. These findings demonstrate a maternal-zygotic effect of *Mbtps1*, to our knowledge the second mammalian gene to which such an effect has been ascribed. Mouse *Zfp57* was the first identified mammalian maternal-zygotic effect gene and was found to participate in the maintenance of genomic DNA methylation imprints without which mouse embryos died in midgestation (Li *et al.* 2008). ZFP57, together with its cofactor KAP1, recruits DNA methyltransferases to a methylated hexanucleotide within numerous imprinting control regions (Quenneville *et al.* 2011; Zuo *et al.* 2011). A role for S1P in genomic imprinting remains to be tested.

Experiments in which *Drosophila* was used support an evolutionarily conserved requirement for the function of the S1P/S2P module during embryonic development. Analogously to *wrt* mutant mice, *dS2P* homozygous mutant fly embryos from heterozygous mothers emerged at a normal frequency, whereas less than 50% of the expected number of homozygous offspring derived from homozygous mothers survived (Matthews *et al.* 2009). The survival rate of heterozygous offspring of homozygous female flies was not reported in this study, leaving open the possibility that *dS2P* may function as a maternal-zygotic effect gene. However, in contrast to the nonredundant function of S1P in mice, the caspase drICE can partially compensate for dS2P deficiency in *Drosophila*, thus permitting a significant proportion of homozygous fly embryos derived from homozygous mothers to survive to adulthood (Amarneh *et al.* 2009). Notably, maternal effect embryonic lethality of homozygous mutant flies was completely rescued by supplementation of the embryo culture medium with fatty acids (Matthews *et al.* 2009). Fatty acids and/or cholesterol may likewise be critically lacking during development of *Mbtps1^wrt/wrt^* concepti *in utero*.

We found that cholesterol and lipoproteins were preferentially reduced relative to triglycerides in serum from *Mbtps1^wrt/wrt^* mice, an effect that we attribute to a more severe impairment of SREBP2 processing as compared with SREBP1 processing. Y496, the residue mutated in *woodrat* mice, may provide a contact critical for interaction with SREBP2.

## References

[bib1] Amarneh B. Matthews K. A. Rawson R. B., 2009 Activation of sterol regulatory element-binding protein by the caspase drice in drosophila larvae. J. Biol. Chem. 284: 9674–9682 19224859 10.1074/jbc.M900346200 PMC2665088

[bib2] Beyer W. R. Popplau D. Garten W. von Laer D. Lenz O., 2003 Endoproteolytic processing of the lymphocytic choriomeningitis virus glycoprotein by the subtilase SKI-1/S1P. J. Virol. 77: 2866–2872 12584310 10.1128/JVI.77.5.2866-2872.2003 PMC149737

[bib3] Boissy R. E. Moellmann G. E. Lerner A. B., 1987 Morphology of melanocytes in hair bulbs and eyes of vitiligo mice. Am. J. Pathol. 127: 380–388 3578491 PMC1899747

[bib4] Brandl K. Rutschmann S. Li X. Du X. Xiao N., 2009 Enhanced sensitivity to DSS colitis caused by a hypomorphic Mbtps1 mutation disrupting the ATF6-driven unfolded protein response. Proc. Natl. Acad. Sci. USA 106: 3300–3305 19202076 10.1073/pnas.0813036106 PMC2651297

[bib5] Chakraborty A. K. Funasaka Y. Slominski A. Ermak G. Hwang J., 1996 Production and release of proopiomelanocortin (POMC) derived peptides by human melanocytes and keratinocytes in culture: Regulation by ultraviolet B. Biochim. Biophys. Acta 1313: 130–138 8781560 10.1016/0167-4889(96)00063-8

[bib6] Contois J. H. Gillmor R. G. Moore R. E. Contois L. R. Macer J. L., 1999 Quantitative determination of cholesterol in lipoprotein fractions by electrophoresis. Clin. Chim. Acta 282: 1–14 10340430 10.1016/s0009-8981(98)00186-7

[bib7] Eberle D. Hegarty B. Bossard P. Ferre P. Foufelle F., 2004 SREBP transcription factors: Master regulators of lipid homeostasis. Biochimie 86: 839–848 15589694 10.1016/j.biochi.2004.09.018

[bib8] Ehrmann M. Clausen T., 2004 Proteolysis as a regulatory mechanism. Annu. Rev. Genet. 38: 709–724 15568990 10.1146/annurev.genet.38.072902.093416

[bib9] Hansdottir A. G. Palsdottir K. Favor J. Neuhauser-Klaus A. Fuchs H., 2004 The novel mouse microphthalmia mutations mitfmi-enu5 and mitfmi-bcc2 produce dominant negative mitf proteins. Genomics 83: 932–935 15081122 10.1016/j.ygeno.2003.10.013

[bib10] Hirobe T., 2011 How are proliferation and differentiation of melanocytes regulated? Pigment Cell. Melanoma Res. 24: 462–478 21375698 10.1111/j.1755-148X.2011.00845.x

[bib11] Hirobe T. Takeuchi S. Hotta E., 2004 The melanocortin receptor-1 gene but not the proopiomelanocortin gene is expressed in melanoblasts and contributes their differentiation in the mouse skin. Pigment Cell Res. 17: 627–635 15541020 10.1111/j.1600-0749.2004.00179.x

[bib12] Kieft K. A. Bocan T. M. Krause B. R., 1991 Rapid on-line determination of cholesterol distribution among plasma lipoproteins after high-performance gel filtration chromatography. J. Lipid Res. 32: 859–866 2072044

[bib13] Krude H. Biebermann H. Luck W. Horn R. Brabant G., 1998 Severe early-onset obesity, adrenal insufficiency and red hair pigmentation caused by POMC mutations in humans. Nat. Genet. 19: 155–157 9620771 10.1038/509

[bib14] Kunz S. Edelmann K. H. de la Torre J. C. Gorney R. Oldstone M. B., 2003 Mechanisms for lymphocytic choriomeningitis virus glycoprotein cleavage, transport, and incorporation into virions. Virology 314: 168–178 14517070 10.1016/s0042-6822(03)00421-5

[bib15] Lenz O. ter Meulen J. Klenk H. D. Seidah N. G. Garten W., 2001 The lassa virus glycoprotein precursor GP-C is proteolytically processed by subtilase SKI-1/S1P. Proc. Natl. Acad. Sci. USA 98: 12701–12705 11606739 10.1073/pnas.221447598 PMC60117

[bib16] Lerner A. B., 1986 Vitiligo (vit). Mouse News Lett 74: 125

[bib17] Li X. Ito M. Zhou F. Youngson N. Zuo X., 2008 A maternal-zygotic effect gene, Zfp57, maintains both maternal and paternal imprints. Dev. Cell 15: 547–557 18854139 10.1016/j.devcel.2008.08.014 PMC2593089

[bib18] Matthews K. A. Kunte A. S. Tambe-Ebot E. Rawson R. B., 2009 Alternative processing of sterol regulatory element binding protein during larval development in *Drosophila melanogaster*. Genetics 181: 119–128 19015545 10.1534/genetics.108.093450 PMC2621160

[bib19] Mitchell K. J. Pinson K. I. Kelly O. G. Brennan J. Zupicich J., 2001 Functional analysis of secreted and transmembrane proteins critical to mouse development. Nat. Genet. 28: 241–249 11431694 10.1038/90074

[bib20] Montoliu L. Oetting W. S. Bennett D. C., 2011 Color Genes. European Society for Pigment Cell Research. Available at: http://www.espcr.org/micemut

[bib21] Murakami T. Kondo S. Ogata M. Kanemoto S. Saito A., 2006 Cleavage of the membrane-bound transcription factor OASIS in response to endoplasmic reticulum stress. J. Neurochem. 96: 1090–1100 16417584 10.1111/j.1471-4159.2005.03596.x

[bib22] Patra D. Xing X. Davies S. Bryan J. Franz C., 2007 Site-1 protease is essential for endochondral bone formation in mice. J. Cell Biol. 179: 687–700 18025304 10.1083/jcb.200708092 PMC2080931

[bib23] Pears J. S. Jung R. T. Bartlett W. Browning M. C. Kenicer K., 1992 A case of skin hyperpigmentation due to alpha-MSH hypersecretion. Br. J. Dermatol. 126: 286–289 1313279 10.1111/j.1365-2133.1992.tb00660.x

[bib24] Popkin D. L. Teijaro J. R. Sullivan B. M. Urata S. Rutschmann S., 2011 Hypomorphic mutation in the site-1 protease Mbtps1 endows resistance to persistent viral infection in a cell-specific manner. Cell Host Microbe 9: 212–222 21402360 10.1016/j.chom.2011.02.006 PMC3058147

[bib25] Quenneville S. Verde G. Corsinotti A. Kapopoulou A. Jakobsson J., 2011 In embryonic stem cells, ZFP57/KAP1 recognize a methylated hexanucleotide to affect chromatin and DNA methylation of imprinting control regions. Mol. Cell 44: 361–372 22055183 10.1016/j.molcel.2011.08.032 PMC3210328

[bib26] Raggo C. Rapin N. Stirling J. Gobeil P. Smith-Windsor E., 2002 Luman, the cellular counterpart of herpes simplex virus VP16, is processed by regulated intramembrane proteolysis. Mol. Cell. Biol. 22: 5639–5649 12138176 10.1128/MCB.22.16.5639-5649.2002 PMC133973

[bib27] Rousseau K. Kauser S. Pritchard L. E. Warhurst A. Oliver R. L., 2007 Proopiomelanocortin (POMC), the ACTH/melanocortin precursor, is secreted by human epidermal keratinocytes and melanocytes and stimulates melanogenesis. FASEB J. 21: 1844–1856 17317724 10.1096/fj.06-7398com PMC2253185

[bib28] Sakai J. Rawson R. B. Espenshade P. J. Cheng D. Seegmiller A. C., 1998 Molecular identification of the sterol-regulated luminal protease that cleaves SREBPs and controls lipid composition of animal cells. Mol. Cell 2: 505–514 9809072 10.1016/s1097-2765(00)80150-1

[bib29] Slominski A. Tobin D. J. Shibahara S. Wortsman J., 2004 Melanin pigmentation in mammalian skin and its hormonal regulation. Physiol. Rev. 84: 1155–1228 15383650 10.1152/physrev.00044.2003

[bib30] Stirling J. O’hare P., 2006 CREB4, a transmembrane bZip transcription factor and potential new substrate for regulation and cleavage by S1P. Mol. Biol. Cell 17: 413–426 16236796 10.1091/mbc.E05-06-0500 PMC1345678

[bib31] Vincent M. J. Sanchez A. J. Erickson B. R. Basak A. Chretien M., 2003 Crimean-congo hemorrhagic fever virus glycoprotein proteolytic processing by subtilase SKI-1. J. Virol. 77: 8640–8649 12885882 10.1128/JVI.77.16.8640-8649.2003 PMC167219

[bib32] Wakamatsu K. Graham A. Cook D. Thody A. J., 1997 Characterisation of ACTH peptides in human skin and their activation of the melanocortin-1 receptor. Pigment Cell Res. 10: 288–297 9359624 10.1111/j.1600-0749.1997.tb00688.x

[bib33] Yang J. Goldstein J. L. Hammer R. E. Moon Y. A. Brown M. S., 2001 Decreased lipid synthesis in livers of mice with disrupted site-1 protease gene. Proc. Natl. Acad. Sci. USA 98: 13607–13612 11717426 10.1073/pnas.201524598 PMC61088

[bib34] Ye J. Rawson R. B. Komuro R. Chen X. Dave U. P., 2000 ER stress induces cleavage of membrane-bound ATF6 by the same proteases that process SREBPs. Mol. Cell 6: 1355–1364 11163209 10.1016/s1097-2765(00)00133-7

[bib35] Zhang K. Shen X. Wu J. Sakaki K. Saunders T., 2006 Endoplasmic reticulum stress activates cleavage of CREBH to induce a systemic inflammatory response. Cell 124: 587–599 16469704 10.1016/j.cell.2005.11.040

[bib36] Zuo X. Sheng J. Lau H. T. McDonald C. M. Andrade M., 2012 Zinc finger protein ZFP57 requires its co-factor to recruit DNA methyltransferases and maintains DNA methylation imprint in embryonic stem cells via its transcriptional repression domain. J. Biol. Chem. 287: 2107–2118 22144682 10.1074/jbc.M111.322644 PMC3265890

